# Clinical Features in Patients With PANDAS/PANS and Therapeutic Approaches: A Retrospective Study

**DOI:** 10.3389/fneur.2021.741176

**Published:** 2021-09-28

**Authors:** Isabella Rea, Cristiana Alessia Guido, Alberto Spalice

**Affiliations:** Child Neurology Division, Department of Pediatrics, “Sapienza” University of Rome, Rome, Italy

**Keywords:** pediatric acute-onset neuropsychiatric syndrome (PANS), pediatric autoimmune neuropsychiatric disorders associated with streptococcal infection (PANDAS), obsessive compulsive disorder, tics, pediatrics, streptococcus beta hemolytic

## Abstract

**Objective:** The clinical characteristics of patients with PANDAS (pediatric autoimmune neuropsychiatric disorders associated with streptococcal infection) and PANS (pediatric acute-onset neuropsychiatric syndrome) and the efficacy of antibiotic therapy with psychotherapy and antipsychotics were investigated to improve neurological symptoms as well as obsessive compulsive disorder (OCD).

**Methods:** We retrospectively analyzed 62 patients with a clinical diagnosis of PANDAS/PANS enrolled from May 14, 2013 to September 15, 2020 in the Neurology Childhood Division, Department of Pediatrics at Sapienza, Rome. Clinical manifestations, neurological and psychiatric, laboratory investigations, and familiar history were collected to evaluate the differences between the two groups. The effects of various therapeutic approaches were examined. Descriptive and comparative statistical analyses were performed.

**Results:** The mean age at onset of PANDAS/PANS symptoms was 6.2 ± 1.2 years. The most common diagnosis was PANDAS, followed by PANS. Neurological and psychiatric symptoms were mostly evident in both groups (>70% of the population), with no significant difference between them (*P* = 0.52 and *P* = 0.15, respectively). Irritability, aggressivity, and food restriction were more prevalent in children with PANS than in those with PANDAS (*P* = 0.024 and *P* = 0.0023, respectively). The levels of anti-streptolysin O and anti-DNAse B 10-fold higher in PANDAS than those in PANS (*P* < 0.0001). Antibiotics or psychotherapy were administered in most cases (90.3 and 53.2%, respectively), followed by antipsychotic treatments (24.2%). In the multivariate analysis, among the therapies used, psychotherapy significantly resulted in the most efficacious relief of OCD, reducing stress in patients and their parents (*P* = 0.042).

**Conclusion:** Our findings confirm a clear clinical difference between the two groups, PANDAS and PANS, using different approaches. In fact, irritability, aggressivity, and food restriction were significantly more frequent in children with PANS and the levels of anti-streptolysin O and anti-DNAse B were higher in PANDAS. Another relevant finding is the efficacy of psychotherapy, especially for obsessive-compulsive disorder, and of antibiotic prophylaxis in managing acute neurological symptoms.

## Introduction

When first defined in 1998 (1), pediatric autoimmune neuropsychiatric disorders associated with streptococcal infection (PANDAS) described a group of 50 children with sudden onset of obsessive-compulsive disorder (OCD) and tic disorders triggered by group A beta-hemolytic streptococcal (GABHS) infection. These children also revealed a complex constellation of symptoms such as personality change, separation anxiety, emotional lability, deterioration in handwriting, and somatic symptoms, including insomnia, urinary frequency, and enuresis. Subsequently, PANDAS has had a controversial diagnosis on clinical and autoimmune bases because of the difficulty in finding a link between streptococcal infection and the onset of neuropsychiatric symptoms (2). Thus, a new clinical entity named pediatric acute-onset neuropsychiatric syndrome (PANS), characterized by abrupt-onset OCD and/or restricted food intake with concurrent presence of neuropsychiatric symptoms such as anxiety, depression, and irritability, was proposed to be triggered by various etiological agents of infection. These activate the immune response and release of chemical mediators of inflammation at the CNS level (3–6). Therefore, PANDAS diagnosis is considered a subset of PANS.

Currently, the pathophysiology is not yet clearly understood, due to the lack of a definitive proof of concept of the autoimmune components. One of the major proposals is based on molecular mechanisms in children with PANDAS, such as the production of antibodies against streptococcal proteins that find brain proteins as targets, most of all in the basal ganglia (7), or antibodies that bind to striatal cholinergic interneurons, inducing rapid-onset OCD symptoms (8).

Clinical and pathophysiological definitions of PANDAS and PANS are crucial for children to receive specific and helpful therapy. These patients may benefit from antibiotics to counteract the source of neuroinflammation, immunomodulatory treatments to regulate the immune system, and psychiatric medications to provide symptomatic relief (9).

The objective of the present study was to describe the clinical, biological, and psychological features of a group of patients with clinical criterias of PANDAS or PANS referred to our hospital. During their course, the patients were subjected to different therapies; therefore, in a retrospective analysis, we compared the efficacy of different therapeutic approaches to improve their health.

## Materials and Methods

### Study Design

Patients aged between 3 and 24 (mean average 9.6 ± 1.1) years who visited the Neurology Department of Sapienza University between May 14, 2013 and September 15, 2020 were included in this study. The patients consisted of 15 children with a clinical diagnosis of PANS using the clinical criteria described by Swedo et al. (3), and 47 children with a clinical diagnosis of PANDAS using Swedo's diagnostic criteria (1). Almost all patients had vocal or motor tics (58/62, 93.5%) and OCD (49/62, 79%).

Patients excluded from the retrospective study were those with neurological pathologies different from PANDAS/PANS (simple tics, ADHD, pathologies of the acoustic spectrum, cognitive retardation, and chronic neurological pathologies).

A follow-up for the efficacy of therapy (antibiotics, psychotherapy, or antipsychotics) was conducted. No informed consent was requested because it was a non-interventional study design.

### Clinical History and Laboratory Tests

At the first evaluation, the medical history, including symptoms at disease onset, exposure to infections, episodes of fever, and the presence of psychiatric disorders together with family history were collected.

Physical and neurological examinations were performed by a child neuropsychiatrist with expertise in movement and psychiatric disorders.

Laboratory tests for infection included anti-streptolysin O (ASO) and anti-deoxyribonuclease B (anti-DNAse B). Laboratory thresholds that suggested streptococcal infection were 200 international units (IU)/mL for ASO and 150 IU/mL for anti-DNAse B.

Psychological evaluation was performed by an expert psychologist using age-standardized tests (Table 1) (10–13).

**Table 1:** Neuropsychological tests.

**Function**	**Test**	**Age range and test description**
Motor and vocal tics	YGTSS	6–24 years- Presence and severity of motor and vocal tics
Obsession and compulsion	CYBOCS	6–24 years- Presence and severity of child's obsession and compulsion

*YGTSS, Yale Global Tic Severity Scale; CYBOCS, Children's Yale-Brown Obsessive Compulsive Scale*.

### Therapies

To counteract neurological and psychiatric symptoms, almost all patients received different antibiotics delivered according to different therapeutic schemes, including azithromycin followed by penicillin, amoxicillin-clavulanic acid followed by benzylpenicillin and azithromycin, amdinocillin followed by cephalosporin, and clarithromycin. Half of the patients followed a program of psychotherapy, whereas only a minor population was treated with antipsychotics such as haloperidol, risperidone, aripiprazole, clozapine, methylphenidate, and pimozide.

### Statistical Analysis

Data are described as mean and standard error or median and range for continuous variables, and as absolute and relative frequencies for categorical variables. A non-parametric analysis (Mann–Whitney *U*-test) for continuous variables and the chi-square test for categorical variables were used to measure differences between groups. *P* ≤ 0.05 was considered statistically significant. Statistical analysis was performed using SPSS for Windows (SPSS Inc., Chicago, IL, USA).

## Results

### Clinical Features and Epidemiology

Sixty-two children (49 men and 13 women) were included in the study according to the clinical diagnosis. Only 15 children were diagnosed with PANS, whereas 47 children were diagnosed with PANDAS. Clinical data summarized in Table 2 showed that the highest symptoms were neurological and OCD, with no statistical difference between the two groups (*P* = 0.52 and *P* = 0.15, respectively), although all PANS children presented OCD completely (15/15 = 100%).

**Table 2:** Clinical and epidemiological features.

	**PANDAS *N* = 47 (%)**	**PANS*N* = 15 (%)**	**PANDAS vs. PANS *P*-value**
Tics	45 (95.7)	13 (86.7)	0.52
Obsessive-compulsive symptoms	34 (72.3)	15 (100)	0.15
Worsening of school performance	8 (17)	3 (20)	0.79
Anxiety	10 (21.2%)	5 (33.3)	0.74
Irritability and aggressivity	9 (19.1)	8 (53.3)	**0.024**
Food restriction	0 (0)	4 (26.7)	**0.0023**

*PANDAS, pediatric autoimmune neuropsychiatric disorders associated with streptococcal infections; PANS, pediatric acute-onset neuropsychiatric syndrome*.

Other minor neuropsychiatric problems were worsening of school performance and anxiety, which were not statistically different (*P* = 0.79 and 0.74, respectively). Irritability, aggressivity, and food restriction were mainly evident in PANS (*P* = 0.24 and *P* = 0.0023, respectively) (Table 2).

The age at onset of PANDAS and PANS symptoms (mean and standard error) was similar (6.2 and 6.0 years), as reported in Table 3. The ASO value and anti-DNAse B were significantly higher in patients with PANDAS than in PANS (*P* < 0.0001), thus showing a 10-fold higher positivity to A beta-hemolytic Streptococcus (Table 3).

**Table 3:** Age at onset and laboratory tests.

**Patients**	**Age at onset (years)**	**ASO (IU/mL)Mean ± SE**	**Median (rank)**	**Anti-DNAse B (U/mL)Mean ± SE**	**Median (rank)**
PANDAS*N* = 47	6.2 ± 1.1	728 ± 73	600 (50–2,780)	820 ± 191	569 (137–2,693)
PANS*N* = 15	6.0 ± 1.0	102 ± 24	68 (20–261)	93 ± 31	60 (23–240)
*P*-value	1.2	**<0.0001**		**<0.0001**	

*ASO, anti-streptolysin O; PANDAS, pediatric autoimmune neuropsychiatric disorders associated with streptococcal infection; PANS, pediatric acute-onset neuropsychiatric syndrome*.

Most relatives, including parents, siblings, grandparents, and uncles, of the 62 patients reported the presence of neuropsychiatric disorders (45% in total PANDAS and PANS), including bipolar disorder and OCD, intellectual disability, schizophrenia, specific learning disorder (SLD), anxiety disorders, language delay, and post-partum depression symptoms, followed by metabolic disorders (21% in total), that included diabetes and thyroid changes, and cardiovascular pathologies (11%), including hypertension and heart attack. A high percentage of parents with metabolic disorders was evident in the PANS group compared to the PANDAS group (*P* = 0.037; Table 4).

**Table 4:** Clinical features of PANDAS-PANS relatives.

**Family history (disorders)**	**PANDAS *N* = 47 (%)**	**PANS*N* = 15 (%)**	**PANDAS vs. PANS *P*-value**
Psychiatric	19 (40)	9 (60)	0.18
Cardiovascular	4 (8.5)	3 (20)	0.22
Metabolic	7 (14.8)	6 (40)	**0.037**

*Psychiatric disorders included bipolar disorder, OCD, intellectual disability, schizophrenia, specific learning disorders (SLD), anxiety disorders, language delay, and postpartum depression. Cardiovascular pathologies included hypertension and heart attack. Metabolic disorders included diabetes and thyroid alterations*.

### Therapy in PANDAS-PANS Groups

Antibiotic therapy was the most administered therapy in 56/62 patients (90.3% of the population). In particular, this included cycles of oral antibiotic therapy followed by intramuscular drugs every 21 days (that is, azithromycin and subsequent penicillin, amoxicillin-clavulanic acid or azithromycin followed by benzylpenicillin, amdinocillin followed by cephalosporin, and clarithromycin).

Antibiotic therapy did not induce significant benefits on tics (*P* = 0.50) and OCD (*P* = 0.64), although, chi-square test revealed a total or partial beneficial acute effect for some month on both tics and OCD (Table 5).

**Table 5:** Therapy in PANDAS-PANS children.

**Therapy**	**PANDAS-PANS *N* = 62**	**%**	**Tic benefits *P*-value**	**OCD benefits*P*-value**
Antibiotics	56	90.3	0.50	0.64
Antipsychotics	15	24.2	0.60	0.78
Psychotherapy	33	53.2	0.66	**0.042**

Symptomatic therapy, such as haloperidol, risperidone, aripiprazole, clozapine, methylphenidate chloridrate, and pimozide, was administered to a small number of patients (15/62; 24.2%). This type of therapy did not have a significant effect on tics (*P* = 0.60) or neuropsychiatric symptoms (*P* = 0.78).

Psychotherapy was administered to 33/62 children in terms of cognitive behavioral therapy (CBT) techniques, including psychoeducation and exposure with response prevention (ERP). Results after at least 1 year of therapy revealed a significant decrease in OCD symptom severity (*P* = 0.042), but not in tics (*P* = 0.66; Table 5).

## Discussion

The aim of this study was to characterize the clinical, biological, and psychological features of a cohort of patients with PANS and PANDAS. Currently, the relationship between streptococcal infections and neuropsychiatric disorders is a complex question. The diagnosis of PANDAS and PANS in this study was based on clinical criteria, with an exclusionary diagnosis with respect to other neurological and psychiatric disorders, as also reported in the first PANS Consensus Conference of 2013 (14) and according to the diagnostic criteria of patients with PANDAS and PANS (15).

In our study, we found that the highest symptoms in children with PANDAS and PANS were tics and OCD, with no statistical difference between the two groups. Anxiety, worsening in school performance, irritability, and aggressivity were also observed mostly of all in patients with PANS compared to PANDAS.

In particular, PANS revealed significantly higher evidence of irritability, aggressivity, and food restriction. These data confirm what was previously reported by Murphy et al. (16), who found that major symptoms such as irritability, behavioral regression, handwriting deterioration, difficulty in elaboration speed and attention, and somatic symptoms (sleep and urinary disorders) were observed in patients with PANS in addition to the main symptoms (OCD and food restriction).

A link between neuropsychological functions and cortico-subcortical network dysfunction was found in both patients with PANDAS and PANS. In PANDAS serum, the presence of anti-dopaminergic autoantibodies was measured, so dysregulating basal ganglia functions through the impact on cholinergic interneurons (17). In PANS etiology, studies on immune-mediated basal ganglia and striatal involvement in the pathogenesis of psychotic disorders have been reported (18). Inflammation has also been recognized by PET imaging in the basal ganglia and striatum of patients with PANDAS (19), and in the thalamus, basal ganglia, and amygdala in children with PANS (9, 20).

Our results on the onset of symptoms agree with the data available to date. Singer and Loiselle (21) reported a mean age in early childhood (6.3 ± −1.2 years) with a prevalence of male sex in a ratio of ~3:1 for subjects with PANDAS.

Streptococcal infection may represent an environmental trigger for tic disorders, a multifactorial condition with a genetic basis. With regard to the biological aspect, our findings show a significant difference for ASO and anti-DNAse B between the PANDAS and PANS groups, revealing that they are two markers important for identifying subjects with PANDAS. In contrast, Leckman et al. (10) showed little significance for these two biomarkers.

Recently, in the research of markers for diagnosis, an alteration of plasma metabolic profile was observed in patients with PANS compared to healthy children, with regard to the metabolite levels associated with neurotransmission (glycine, tryptophan, histamine/histidine) and neuroinflammation (glutamine, 2-hydroxybutirate) (22).

In our study, taking into account the familiar history, a high percentage of relatives with neuropsychiatric diseases, with respect to metabolic and cardiovascular diseases, was observed. A major percentage of relatives with metabolic disorders, including diabetes and thyroid disorders, was found in children with PANS with respect to PANDAS. In other studies, there were only a few cases of parents with autoimmune diseases; in others, family history played a role with high rates of maternal autoimmunity or family members with OCD/tics or other neurological disturbances (23, 24).

Regarding pharmacological treatment, therapy is aimed at addressing the physical and psychiatric symptoms to reduce OCD and tics or other behaviors that can interfere with their life and school attendance. Streptococcal infections are treated with antibiotics such as amoxicillin, azithromycin, penicillin, and cephalosporins (25). An improvement in tic and OCD was observed with cefdinir therapy, but the differences were not significant (16). In addition, intravenous immunoglobulin did not appear to be an effective treatment for pediatric autoimmune neuropsychiatric disorders associated with streptococcal infection obsessive-compulsive disorder (26). In our case, the antibiotics as the first therapy were useful in acute treatment for some months to decrease both tics and OCD, but with no significant efficacy for a long time. Antipsychotic therapy was not significantly active in tic or OCD. Swedo et al. (9) reported that some severe cases of PANDAS may not respond to drug and behavioral therapy. In such cases, the treatment guidelines described by the members of the PANS Research Consortium in 2017 recommend immunodulating therapies individually or in combination with corticosteroids, rituximab, intravenous immunoglobulin, plasmapheresis, and mycophenolate (27, 28). Steroids used to improve OCD symptoms can also worsen tics; thus, at present, they are not recommended for the treatment of PANDAS. In a study by Melamed et al. (29), the infusion of intravenous immunoglobulin was able to ameliorate psychological symptoms for at least 8 weeks in 20 subjects with moderate to severe PANS.

In our investigation, patients with at least 1 year of psychotherapy showed a significant decrease in OCD symptom severity, but not in tics. These results confirm those already reported in two studies (30, 31), where psychotherapy as CBT in patients with PANDAS and PANS showed a significant reduction in OCD symptom severity.

In a survey carried out in 473 out of 698 patients receiving some forms of psychotherapy (32), exposure to response prevention (ERP) or CBT was reported to be very effective to treat OCD in 39% of cases.

Other case reports have suggested the use of CBT techniques, including psychoeducation and ERP, in patients with PANDAS and in children with PANS to improve symptomatology (4, 33).

On the whole, the main limitation of our study is represented by the relatively small number of patients studied, although the strength of the investigation by the multidisciplinary approach adopted, in order to characterize the clinical, biological, and psychological features of patients with PANS and PANDAS allowed us to highlight relevant clinical differences between the two types of patients.

## Data Availability Statement

The raw data supporting the conclusions of this article will be made available by the authors, without undue reservation.

## Ethics Statement

Ethical review and approval was not required for the study on human participants in accordance with the local legislation and institutional requirements. Written informed consent to participate in this study was provided by the participants' legal guardian/next of kin.

## Author Contributions

IR was responsible for the study concept and design, data acquisition, analysis, and interpretation of data. CG performed data acquisition, analysis, and interpretation of data. AS conducted critical revision of the manuscript for intellectual content and supervised the study. All authors contributed to the manuscript and approved the submitted version.

## Funding

All phases of this study were supported by the Department of Pediatrics of the Sapienza University of Rome.

## Conflict of Interest

The authors declare that the research was conducted in the absence of any commercial or financial relationships that could be construed as a potential conflict of interest.

## Publisher's Note

All claims expressed in this article are solely those of the authors and do not necessarily represent those of their affiliated organizations, or those of the publisher, the editors and the reviewers. Any product that may be evaluated in this article, or claim that may be made by its manufacturer, is not guaranteed or endorsed by the publisher.
